# Validation of Instrumented Football Shoes to Measure On-Field Ground Reaction Forces

**DOI:** 10.3390/s22103673

**Published:** 2022-05-11

**Authors:** Alexandre Karamanoukian, Jean-Philippe Boucher, Romain Labbé, Nicolas Vignais

**Affiliations:** 1 CIAMS, Université Paris Saclay, 91405 Orsay, France; nicolas.vignais@universite-paris-saclay.fr; 2 CIAMS, Université d’Orléans, 45067 Orléans, France; 3 Phyling, 91120 Palaiseau, France; jean-philippe.boucher@phyling.fr (J.-P.B.); romain.labbe@phyling.fr (R.L.)

**Keywords:** ground reaction forces, embedded sensors, football, stud, force platform

## Abstract

Ground reaction forces (GRF) have been widely studied in football to prevent injury. However, ambulatory tools are missing, posing methodological limitations. The purpose of this study was to assess the validity of an innovative football shoe measuring normal GRF (nGRF) directly on the field through instrumented studs. A laboratory-based experiment was first conducted to compare nGRF obtained with the instrumented shoe (IS) to vertical GRF (vGRF) obtained with force platform (FP) data, the gold standard to measure vGRF. To this aim, three subjects performed 50 steps and 18 counter-movement jumps (CMJs). Secondly, eleven subjects completed running sprints at different velocities on a football field, as well as CMJs, while wearing the IS. Good to excellent agreement was found between the vGRF parameters measured with the FP and the nGRF measured by the IS (ICC > 0.75 for 9 out of 11 parameters). Moreover, on-field nGRF patterns demonstrated a progressive and significant increase in relation with the running velocity (*p* < 0.001). This study demonstrated that the IS is a highly valid tool to assess vGRF patterns on a football field. This innovative way to measure vGRF in situ could give new insights to quantify training load and detect neuromuscular fatigue.

## 1. Introduction

Football is one of the most popular sports in the world with over 240 million people regularly playing [1] and nearly 130,000 professional players around the world [2]. However, football is also known to have one of the highest injury incidence risks of any sport [3,4,5,6,7]. In male football, most injuries (59–67%) consist of non-contact injuries, muscular strains being predominant [4,8,9]. Indeed, thigh strain is the most frequent injury, particularly in the hamstring (12.8% of all injuries), followed by adductor strain [9]. Moreover, a large number of ligament injuries also occur without any contact with another player, e.g., 70–84% of anterior cruciate ligament (ACL) tears are non-contact [10]. 

Non-contact injuries are more frequent in the second half and towards the end of the match, possibly due to fatigue accumulation [11,12,13,14]. It is also known that players participating in two matches per week have an injury rate up to six times higher than players who compete in only one match per week [7,15]. Furthermore, Gabett stated that “excessive and rapid increases in training loads are likely responsible for a large proportion of non-contact, soft-tissue injuries” [16]. Thus, fatigue and excessive training load have been identified as major risk factors. Moreover, re-injuries (i.e., injury of the same type and location within two months of the previous injury) are predominant in professional football (they account for 22–30% of all injuries), which raises questions about the management of training load in rehabilitation [4,17]. Although fatigue and maladjusted training load are known among researchers and professional coaches/physiotherapists, injury rates for muscle and severe injuries did not decrease during the 2000s—hamstring injuries have even increased during this decade [9,18]. The previous risk factors associated with the increasing number of injuries highlight the fact that a better in-field biomechanical analysis is required to prevent non-contact injuries in football. Consequently, training load monitoring has grown exponentially over the past few years due to the development of devices such as Global Positioning System (GPS), which allow coaches to obtain distance, speed and acceleration of players. However, ground reaction forces (GRF) have greater potential for monitoring players’ fatigue since they accurately provide biomechanical load, impact forces and total impulse data achieved by players [19,20]. Therefore, the use of inertial measurement units (IMUs) to estimate GRF has gained interest among football clubs and federations [21,22]. These devices are currently used by professional clubs to gain better insight on players’ fatigue and training efficiency throughout the season. IMUs are often integrated into GPS devices and used as an indirect measurement tool to compute GRF from complex biomechanical models and the derived acceleration and deceleration signals. These estimations have proven to be reliable in certain basic conditions such as 15 and 30 m sprints [23] but have several drawbacks on the field. Indeed, integrated accelerometers have shown poor or limited validity during team sport activities [20,24], football-specific tasks, [25] and change of direction tasks [21]. Therefore, very few of the available markers characterizing an athletes’ training load have shown strong scientific validity [26]. 

Biomechanical data like GRF have been widely studied in the football research area. However, existing literature failed to find solutions to prevent non-contact injuries. These contradictory results could be explained by several drawbacks. Most of the studies used a mechanical apparatus, i.e., artificial feet which simulated a human step to obtain GRF [27,28,29,30,31,32,33,34,35,36,37]. These devices do not represent the complexity of the human walking and running in the field very well. Moreover, differences exist between mechanical and biomechanical (with subjects) testing protocols [38]. Thus, it is essential to consider the intra- and inter-subject variability when collecting GRF. Other studies used real subjects and standard force platforms (covered with an artificial layer of grass) to obtain GRF on one or two steps [34,39,40,41]. These laboratory-based studies do not reliably replicate the normal playing conditions due to both the small number of steps taken and the fact that they used neither natural nor artificial turf on which players would usually compete. It is paramount to understand that GRF are related to adaptations between the player, his shoes and the surface. Thus, any changes from ecological and usual conditions of play may alter the relevance and validity of the results. 

To conclude, the lack of adequate devices or methods available to measure (or accurately estimate) GRF on a football field may limit injury prevention strategies, which could explain the current increase in non-contact injuries in football.

The recent development of instrumented studs that are capable of recording on-field normal GRF (nGRF) therefore represents a breakthrough. These studs can record nGRF without altering standard conditions of play and can be fitted on any commercially available shoe. Thus, collected nGRF are directly related to the player, his shoes and his movements in any given situation, increasing the ecological relevance of obtained data. 

The aim of this study was to assess the validity of a pair of football shoes fitted with 12 instrumented studs as a device to measure vertical GRF (vGRF) on a football field. To assess this validity, a laboratory-based experiment was first conducted to compare nGRF parameters collected by the instrumented shoe (IS) to force platform (FP) data, i.e., the reference tool to measure vGRF, during running and countermovement jumps (CMJs). A second experiment was conducted on the field to compare data obtained in ecological conditions during sprints and CMJs to data obtained with the force platform from previous studies.

## 2. Materials and Methods

### 2.1. Subjects

Eleven amateur football players (11 males, mean ± SD: age, 22 ± 1.2 y; height, 1.76 ± 0.05 m; body mass, 70 ± 4.9 kg) were recruited. Subjects were selected based upon two criteria: participation in regular football practice (once a month or more) and having no lower limb injury within the 6 months prior to the study. Each subject had a shoe size between US 9 and 9.5 (or EUR 42.5–43) as required by the available experimental shoe (9.5 US/43 EUR). Subjects provided written informed consent before taking part in this study. This study was approved by the academic Research Ethics and Scientific Integrity Council (Université Paris-Saclay, 2021-170) and was conducted in accordance with the Declaration of Helsinki.

### 2.2. Instrumented Stud

**Figure 1:**
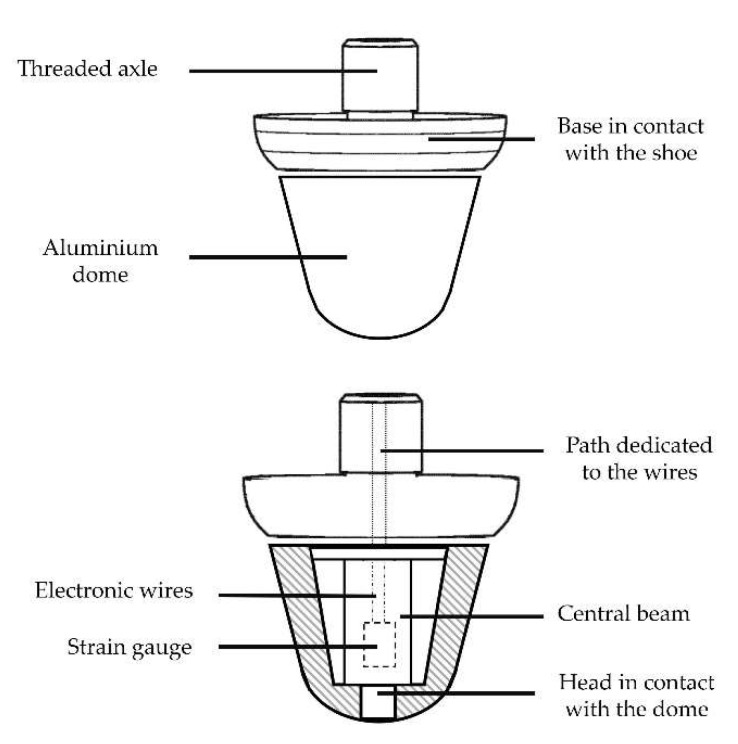
Instrumented stud representation (adapted from Labbé et al., 2018 [42]).

This technology has been adapted from the patent by Labbé et al. [42], as shown in Figure 1. Each instrumented stud was composed of two parts: a base connected to the shoe with a central square body whose deformations were measured with strain gauges and a dome that was connected to the central square body and in contact with the ground. While the patent describes a shoe with instrumented studs measuring forces in 3 directions, the shoe used in this study measured only the forces along the longitudinal axis of the stud using 4 strain gauges stuck around the central square body of the instrumented stud. The wires of each stud entered the shoe below the insole and were connected to a custom-made electronic board at the rear of the shoe.

### 2.3. Materials

**Figure 2:**
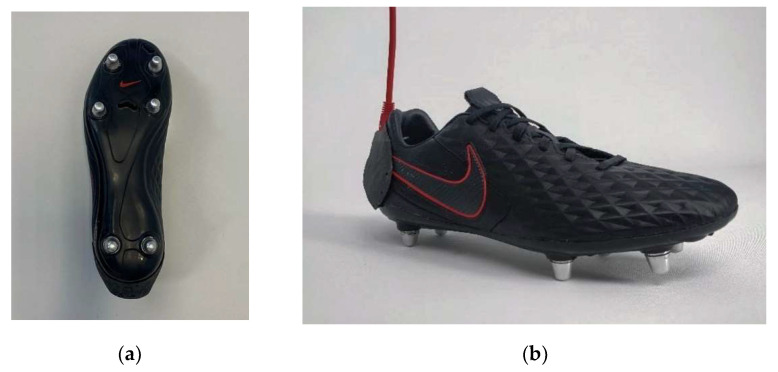
(**a**) Football shoe with six instrumented studs in the transversal plane (**b**) and integrated electronics at the back.

One pair of commercially available football shoes (Tiempo Legend VIII Pro SG, Nike^®^, Beaverton, OR, USA) with removable studs was equipped with 12 instrumented studs (6 on each shoe) as shown in Figure 2. Each stud measured the force along its normal axis (nGRF) in the referential frame related to the shoe using strain gauges located inside them. The total normal force measured by the shoe was obtained by summing the nGRF of each individual stud. These studs were made of aluminum and measured 12 mm. They were located at the heel (lateral and medial), under the 1st and 5th metatarsals and under the 1st and 5th phalanges. The data acquisition system (MaxiPhyling, Phyling, France) was composed of a portable acquisition system (weight = 230 g) worn by subjects in a custom lightweight sport vest (i.e., Apex Vest, STATSport^®^, Newry, UK). Shoes were connected to the data acquisition system using two wires (one per shoe).

The calibration was carried out using a custom-made force platform (Phyling, France) that allowed calibration of each individual stud. This force platform was composed of a large surface and a small central force sensor. The purpose of this force platform was to calibrate each stud by comparing its force data with data from the central force sensor. This was done by targeting the central force sensor with one stud while the five other studs remained on the non-sensitive part of the force platform.

### 2.4. Testing Procedure

#### 2.4.1. Laboratory-Based Experiment

For this part of the experiment, data obtained from the FP during running movements and CMJs was compared to data from the IS. Thus, a force platform (AMTI^®^, Watertown, MA, USA) was covered with artificial turf (FieldTurf, Tarkett^®^, Peachtree City, GA, USA) to allow subjects to run on it in football shoes. The force platform dimensions were 1200 mm × 600 mm. Both the FP and the IS had an acquisition frequency of 800 Hz. For this part of the study, only three subjects took part in the experiment (mean ± SD: age, 23.3 y; height, 176 cm; body mass, 69 kg). After a standardized warm-up, each subject was asked to perform 50 steps with his right foot on the FP and 18 CMJs wearing the instrumented pair of football shoes.

For the running movements, subjects were instructed to run at a self-selected velocity [43] and to land on the FP without adjusting their running pattern. The validity of each trial was controlled by a real-time force-data feedback given to the experimenter.

Subjects were asked to start the CMJs from a standing position with their hands on their hips and then to flex the knees before immediately going upwards to jump vertically. Subjects were given as many trials as needed to familiarize themselves with this jumping technique. They were then required to perform 18 CMJs, with a minimum rest period of 10 s between each jump.

#### 2.4.2. On-Field Experiment

The on-field experiment consisted of CMJs and runs at different velocities. Each subject was required to run in a straight line at 3, 4 and 5 m/s and to perform CMJs. To obtain enough data to consider the intra-subject variability, two runs of 50 m for each velocity and five CMJs were completed by each subject. Cones were placed at 10 m intervals along the 50 m straight line. Each subject was asked to time his run so that he reached the cone at the same time as the audio signal was given (in order to run at the required speed). If the subject was too slow (he was located more than 0.5 m before the cone at the time the signal was given by the speaker) or too fast (he was located more than 0.5 m after the cone at the time the audible signal was given by the speaker), the trial was rejected by the experimenter. Each subject was given a one-minute break between every run and a thirty-second break between each jump. Subjects were allowed to warm up, to familiarize themselves with the running velocities and to become used to the material prior to testing.

**Figure 3:**
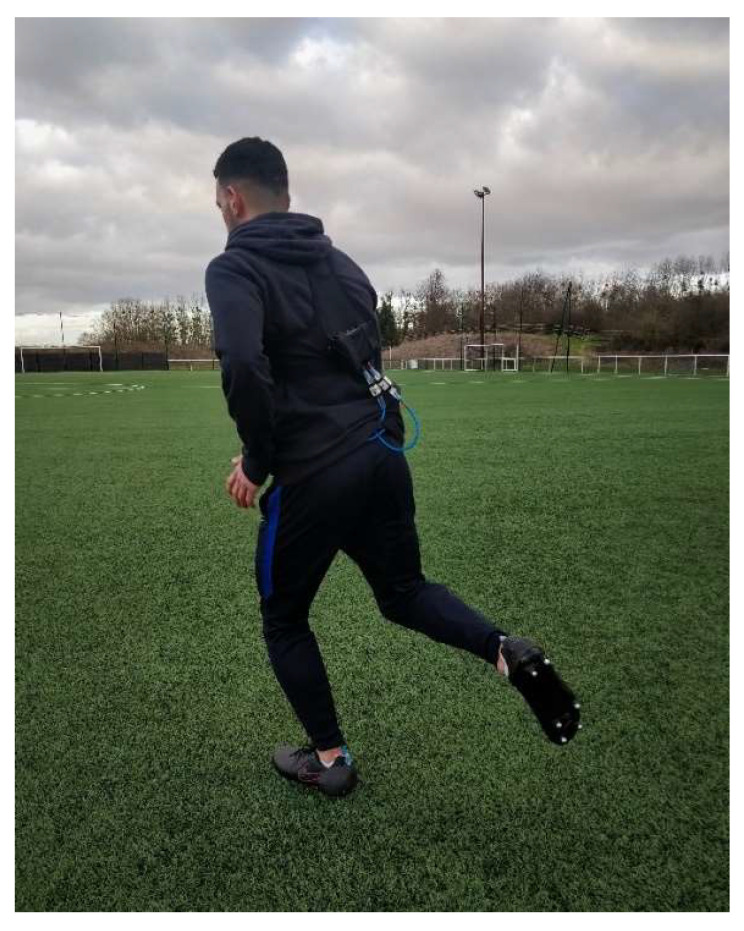
Outdoor football field used in this study and data acquisition system (MaxiPhyling, Phyling, France) connected to each shoe by a wire and worn by subjects in a lightweight sport vest.

The experiment was conducted on an outdoor football field (Figure 3) equipped with synthetic turf (TT+45, EuroField^®^, Aigremont, France). This turf follows the EN 15330-1 and NF P90-112 standards and was in conformity with French Football Federation regulation [44]. Turf moisture was assessed prior to each session to ensure the soil was not too dry or too moist. nGRF were collected at a sampling frequency of 1000 Hz. One of the two instrumented shoes (six studs) was recorded and analyzed during this part of the experiment.

### 2.5. Definition of Reference Frames 

**Figure 4:**
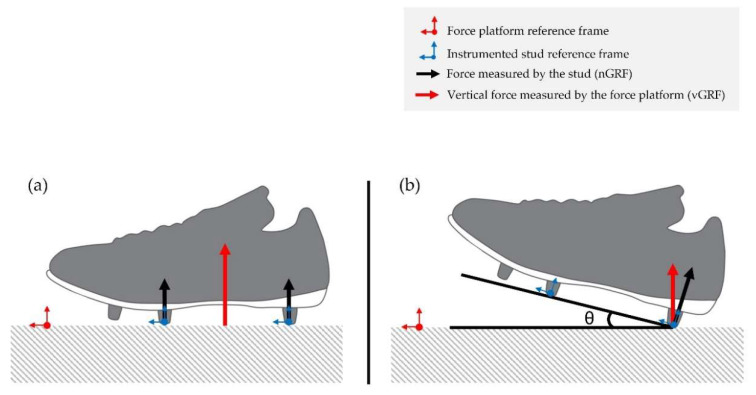
Schematic representation of the forces measured by the instrumented shoe (IS) and the force platform (FP) and their associated reference frames. (**a**) Case where nGRF would be equal to vGRF. (**b**) Case where nGRF would be different to vGRF due to a θ angle at ground strike (adapted from Oudshoorn et al., 2018 [45]).

As previously stated, each stud from the IS measured the force along its normal axis in the referential frame related to the shoe, i.e., nGRF. These studs did not measure shear forces. During the laboratory-based experiment, the nGRF was compared to the vertical component of the GRF given by the FP (vGRF). A schematic representation of the reference frames from the two measurement methods is shown in Figure 4.

### 2.6. Data Analysis

#### 2.6.1. Laboratory-Based Experiment

**Figure 5:**
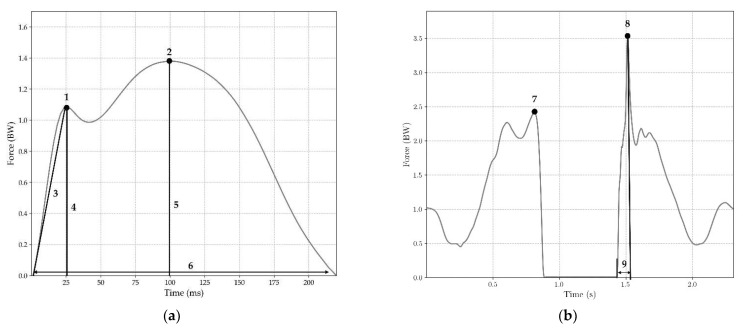
(**a**) Parameters measured for typical running movements: 1 = Fz1, 2 = Fz2, 3 = LR, 4 = tFz1, 5 = tFz2, 6 = CT; (**b**) Parameters measured for CMJs: 7 = FzTO, 8 = FzLan, 9 = tFzLan.

Data analysis was conducted on Python (3.9) and JASP (0.14.1.0). The beginning and end of each step were defined with a 50 N threshold [46]. Raw data were filtered using a low-pass Butterworth filter (100 Hz cutoff, third-order) for both the IS and FP. The mean force-time curve for the 50 steps was computed for the FP and the IS data to allow visual comparison. Kinetics and temporal parameters were extracted for each step and each CMJ according to the definitions presented in Table 1 and Figure 5. Loading Rate (LR) was calculated as the maximal value of the derivative of the vertical GRF (vertical instantaneous loading rate) [47]. Jump height (JH) was computed using flight time, according to the literature [48]. Normalization of force-time data for each subject’s body weight (BW) was conducted to allow inter-subject comparisons.

#### 2.6.2. On-Field Experiment

Relative force-time curve data were computed using time of contact percentages to increase the validity of inter-conditions and inter-subject comparisons. Average force pattern for each subject was computed for each running velocity condition (3, 4 and 5 m/s). The mean force pattern for each velocity was computed by averaging all subjects’ curves for each velocity condition, despite the fact that this method could result in an attenuation of maximum peak values caused by the asynchronous appearances of theses peaks over each subjects’ pattern [46,49].

### 2.7. Statistical Analysis

#### 2.7.1. Laboratory-Based Experiment

To assess the agreement between the IS and FP in the laboratory-based measurements, a two-way mixed model intraclass correlation coefficient (ICC _3,1_) and corresponding 95% interval (CI) were calculated for each parameter. The higher the intraclass coefficient value, the greater the agreement between the two measurements. Values less than 0.50 are indicative of poor reliability, values between 0.50 and 0.75 indicate moderate reliability, values between 0.75 and 0.90 indicate good reliability and values greater than 0.90 indicate excellent reliability, according to the recommendations in existing literature [50]. Agreement between the two measurements was also examined with a Bland Altman plot analysis. This analysis is “a simple way to evaluate a bias between the mean differences, and to estimate an agreement interval, within which 95% of the differences of the second method, compared to the first one, fall” [51]. Limits of agreement (LOAs) were calculated (±1.96 standard deviation of the mean differences between the two measurements) as well as ratio limits of agreement (RLOA) to express LOA as a percentage of mean value of each parameter [52]. 

#### 2.7.2. On-Field Experiment

Mean and standard deviations were calculated for each parameter for each running velocity condition (3, 4 and 5 m/s), as in the study by Hamill et al. [49]. A repeated measures 1 × 7 multivariate analysis of variance (MANOVA) was performed with velocity and nGRF parameters. Correction for the violation of sphericity (Huynh–Feldt) was performed when needed. One-way repeated measures analysis of variance (ANOVA), with a post hoc Bonferroni correction was then conducted on each parameter if significant differences were found. Statistical analysis was conducted using a sample of values (n = 292) from each condition to ensure equal sample sizes among the three conditions. Statistical significance was set at *p* < 0.05.

## 3. Results

### 3.1. Laboratory-Based Experiment

FP and IS mean results and standard deviations for each parameter (n = 50) are presented in Table 2, as well as ICC_(3,1)_ results and LOA.

Results of the statistical analysis showed excellent agreement between the FP and the IS measurements for Fz1, LR, CT, FzLan and JH (ICC values: 0.9–1.0, RLOA values: −4.9–0.2%). Good agreement was found for Fz2, mean force, tFz2 and tFzLan (ICC values > 0.75 and RLOA values: 0.0–4.8%) and moderate agreement was found for FzTO and tFz1 (ICC values > 0.70 and RLOA values: −0.5–11.3%)

**Figure 6:**
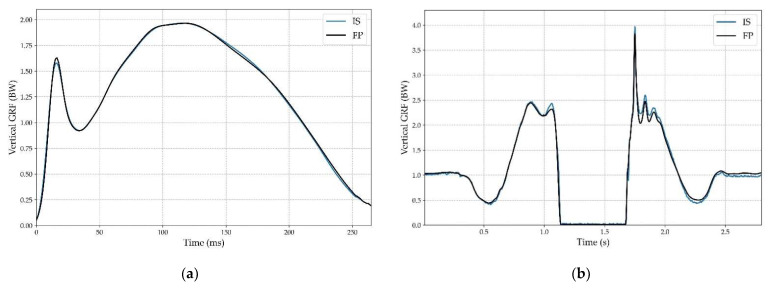
(**a**) Mean vertical ground reaction forces (GRF) vs. time curve computed for the 50 steps by the force platform (FP, black line) and mean normal ground reaction forces (nGRF) vs. time curve by the instrumented shoe (IS, blue line) for one subject; (**b**) Example of a vGRF and a nGRF curve from a CMJ (IS: blue line; FP: black line).

Mean force pattern curve average upon the 50 running steps for each measurement method for one subject and an example of one CMJ force curve are presented in Figure 6. 

Regarding running patterns, results demonstrated very similar curves. CMJ patterns also showed great accordance between the two measurement methods except for a few overestimations from the IS, particularly at the end of the take-off phase.

### 3.2. On-Field Experiment

#### 3.2.1. Running Movements

The total number of steps analyzed for all subjects was 446 for the 3 m/s condition, 393 for the 4 m/s condition and 292 for the 5 m/s condition. Results for each condition are presented in Table 3. Fz1 (and consequently, the corresponding tFz1) was not computed among the three subjects who demonstrated midfoot strike patterns—strike patterns characterized by touching the ground with studs located under the 1st and 5th metatarsals before the studs located at the heel.

The MANOVA showed statistically significant differences between running velocities (F = 73.72; *p* < 0.001). One-way repeated measures ANOVA revealed a significant increase of LR and mean force for all velocity conditions (*p* < 0.001). Significant increases were also found for Fz1 and Fz2 between the 3 m/s and the 5 m/s conditions (*p* < 0.001) but not between the 4 m/s and 5 m/s conditions (*p* = 0.17 for Fz1 and *p* = 0.59 for Fz2). Regarding the temporal parameters, significant decreases were found for tFz1, tFz2 and CT for all running velocities (*p* < 0.001).

**Figure 7:**
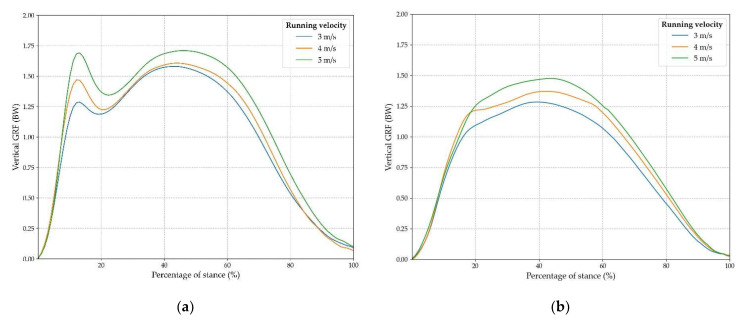
Mean normal GRF curves against percentage of contact time for each velocity condition for rearfoot runners (**a**) and midfoot runners (**b**).

Mean curves for each velocity condition are presented in Figure 7. Normal GRF are expressed against percentage of contact time to allow for inter-condition and inter-subject comparison, as stated previously.

#### 3.2.2. Countermovement Jumps

Due to technical issues (one of the wires passing through the outsole was damaged during part of the experiment), results of CMJS were analyzed for only eight of the eleven subjects. Therefore, a total of 40 CMJs were analyzed in this part of the study. Mean nGRF parameters for these CMJs are presented in Table 4. As stated previously (Section 2.4.2), data presented in Table 4 are from one instrumented shoe only.

## 4. Discussion

The aim of this study was to compare obtained nGRF data with IS to FP data—the gold standard to measure vGRF—in order to assess the validity of the IS. To the best of our knowledge, the current study is the first to collect nGRF directly on a football field with a large number of steps and without altering standard conditions of play. 

Results demonstrated a very strong similarity between the FP and the IS measurements during the laboratory-based study. Similarly, results from the on-field experiment revealed a high degree of accordance with the existing literature [49,53,54,55].

Firstly, the laboratory-based experiment showed strong agreement between the two measurement methods for all parameters. Good to excellent intraclass correlation coefficients were found for force parameters (except for FzTO) as well as for temporal parameters (except for tFz1), according to Koo et al. [50]. The low ratios of limits of agreement also highlight the fact that data from both disposals are similar. Thus, the IS may be considered a highly valid tool to measure vGRF. 

More precisely, with regard to the running movements during the laboratory experiment, the GRF curve measured by the IS revealed some small differences for the impact-related parameters (Fz1, tFz1 and LR) in comparison to parameters measured by the FP. This could be explained by the fact that the studs measured nGRF in their own reference frame and that these values were compared to vGRF measured by the FP, as previously explained (Section 2.5). Consequently, during the impact phase, if a subject strikes the ground without a totally flat-foot position (i.e., a rearfoot or a forefoot runner), nGRF values would be different from vGRF values. In the future, the use of a marker-based motion capture system to determine the foot angle at ground strike could help verify this hypothesis. The pattern of CMJs demonstrated great accordance with the literature, showing a typical double peak in the take-off phase followed by a large landing force peak [56,57].

Secondly, results from the on-field experiment revealed a logical and significant increase of nGRF kinetic parameters in relation to running velocity. Subsequently, mean nGRF profiles showed great accordance with existing studies which measured vGRF on force platforms for similar running velocities [49,53,54,55]. However, the values of some parameters reported in this study were lower than in the literature. Indeed, Fz1 values were located between 1.39 and 1.76 BW (Table 3), whereas Cavanagh et al. reported a first peak above 2 BW [46] and Munro et al. found first peak values ranging from 1.57–2.32 BW for similar velocities [54]. This could be explained by higher shock absorption properties of the real football turf environment in comparison to the hard surfaces of force platforms employed in previous studies. Indeed, artificial turf shock absorption values must be located between 55–70% to comply with international regulations [58], whereas shock absorption values for track surfaces range from 35–50% [59] and asphalt or concrete values are below 2% [60]. Fz2 and LR values were also lower than those reported by Cavanagh et al. but were in accordance with Munro et al.’s findings (LR values: from 80.01–137.07 BW/s in the current study vs. 77.2–113 BW/s).

Normal GRF profiles relating to velocity were similar to patterns reported by Hamill et al., demonstrating an increase of Fz1 and Fz2 peaks in relation with running velocity [49]. The less pronounced first nGRF peak observed among midfoot strikers (Figure 7a) is in accordance with previous studies, with the typical force pattern of this type of runner being known for its absence of an initial first peak [46].

CMJ results obtained during the on-field experiment demonstrated a jump height similar to subjects with the same game level [61]. However, FzLan magnitude in the current study was lower (1.77 BW) than in the existing literature (6.0–9.9 BW) [62,63]. These differences could also be explained by shock absorption differences between the turf used in this study and the force platform used in the literature, as previously stated [58,59,60]. 

The IS may therefore be considered as a valid and reliable tool to directly measure vGRF on the field. As a perspective, this tool may be used to help coaches to efficiently monitor athletes’ training load. Thus, performance analysts might be able to prevent overuse and non-contact injuries by minimizing the accumulation of players’ acute and chronic fatigue. It may also help physiotherapists to more efficiently manage rehabilitation and return-to-play mechanisms, thus reducing probability of re-injury. Additionally, this innovative tool could be added to existing training monitoring metrics (distance covered, number of accelerations, etc.) to assess neuromuscular/running efficiency for example [20].

Although the results demonstrated great accordance between the two measurement methods, it is important to consider some limitations of the current study. Firstly, during the on-field experiment, some non-studded parts of the shoe (such as the outsole of the shoe) may have touched the ground. This may have caused a reduction in the force measured by the IS. However, higher forces have been obtained through the field study, compared to the laboratory study, which tempers this hypothesis. Another limitation of the current study was that fewer subjects (n = 3) participated in the laboratory-based experiment. However, this part of the study considered methodological comparisons rather than subject-related comparisons. Finally, running velocities during the on-field experiment relied solely upon acoustic signals given to the subjects—the use of infrared timing gates would have allowed for more accurate velocity measurements.

In the future, this new technology could also be compared to in-shoe pressure systems, although these systems are known for their relative accuracy in force data measurement [64,65,66] due to the complex environment created by the shoe (i.e., temperature variability, creasing of sensor matrix).

## 5. Conclusions

This study aimed to assess the validity of an instrumented pair of football shoes as a device to measure vGRF. The results demonstrated that this tool is a highly valid device to obtain on-field vGRF. This innovative way of measuring vGRF represents a major development in this area of study, offering remarkable insight to coaches and physiotherapists to help quantify training load and detect stride imbalances.

## 6. Patents

Labbé R., Boucher J.-P., Blanchard S. (2018). *Instrumented studded shoe* (FRFR3086511A1). France. https://patents.google.com/patent/FR3086511A1/en, accessed on 28 September 2018.

## Figures and Tables

**Table 1:** Description of kinetic and temporal vGRF parameters computed during running and countermovement jumps (CMJs).

Movement	Parameter	Description
Running	Fz1	Initial peak of the vertical GRF, normalized to BW.
	Fz2	Propulsive peak of the vertical GRF, normalized to BW.
	LR	Loading Rate, calculated as the maximal value of the derivative of the vertical GRF, in BW/s.
	Mean force	Mean vertical force value, normalized in BW.
	tFz1	Time from foot strike to Fz1 peak, in ms.
	tFz2	Time from foot strike to Fz2 peak, in ms.
	CT	Contact Time, time from foot strike to toe-off, in ms.
CMJ	FzTO	Maximal value of vertical GRF during the take-off phase of the CMJ, normalized to BW.
	FzLan	Maximal value of the vertical GRF during the landing phase of the CMJ, normalized to BW.
	tFzLan	Time from landing phase ground contact to FzLan, in ms
	JH	Jumping Height during the CMJ, in cm.

**Table 2:** Force platform (FP) and instrumented shoe (IS) mean results with standard deviations, intraclass correlations (ICC) values, limits of agreement (LOA) and ratio of limits of agreement (RLOA) for running and countermovement jumps (CMJs).

Movement	Parameter	FP Mean ± SD	IS Mean ± SD	ICC _(3,1)_ [95% CI]	Mean Diff (LOA) (Lower Lim, Upper Lim)	RLOA (%)
Running	Fz1 (BW)	1.52 ± 0.32	1.45 ± 0.31	0.97 [0.96, 0.98]	−0.06 (−0.2,0.08)	−4.3
	Fz2 (BW)	2.02 ± 0.09	2.05± 0.11	0.89 [0.86, 0.92]	0.02 (−0.07, 0.12)	1.2
	LR (BW/s)	145.63± 38.3	138.72 ± 34.65	0.97 [0.96, 0.98]	−6.92 (−23.8, 9.94)	−4.9
	Mean force (BW)	1.26 ± 0.05	1.25 ± 0.05	0.86 [0.81, 0.89]	0.00 (−0.06, 0.05)	−0.5
	tFz1 (ms)	16.38 ± 2.13	16.30 ± 2.37	0.73 [0.65, 0.80]	−0.08 (−3.30, 3.13)	−0.5
	tFz2 (ms)	114.6 ± 10.5	114.6 ± 10.9	0.79 [0.73, 0.85]	0.00 (−13.55, 13.56)	0.0
	CT (ms)	278.59 ± 13.82	279.15 ± 13.3	0.97 [0.96, 0.97]	0.55 (−5.48, 6.60)	0.2
CMJ	FzTO (BW)	2.44 ± 0.18	2.73 ± 0.24	0.70 [0.51, 0.83]	0.29 (−0.03, 0.61)	11.3
	FzLan (BW)	3.20 ± 0.51	3.18 ± 0.52	0.94 [0.90, 0.97]	−0.03 (−0.37, 0.31)	−0.9
	tFzLan (ms)	80.17 ± 15.58	84.14 ± 15.37	0.81 [0.68, 0.89]	−3.97 (−14.58, 22.53)	4.8
	JH (cm)	30.15 ± 4.03	29.89 ± 4.02	1.00 [1.00, 1.00]	−0.26 (−0.55, 0.04)	−0.9

**Table 3:** Mean nGRF and temporal parameters for each condition and corresponding F-ratios. LR = loading rate; CT = contact time; BW = body weight.

Parameter	3 m/s	4 m/s	5 m/s	F-Ratio
Fz1 (BW)	1.39 ± 0.44	1.66 ± 0.49	1.76 ± 0.55	19.24 ***
Fz2 (BW)	1.46 ± 0.39	1.64 ± 0.49	1.68 ± 0.49	19.40 ***
LR (BW/s)	80.01 ± 32.27	109.08 ± 39.46	137.07 ± 57.93	120.60 ***
Mean (BW)	0.99 ± 0.21	1.12 ± 0.27	1.14 ± 0.27	31.96 ***
tFz1 (ms)	32.13 ± 13.27	25.94 ± 5.57	22.05 ± 6.18	35.33 ***
tFz2 (ms)	89.92 ± 22.33	80.98 ± 20.23	66.22 ± 17.47	96.20 ***
CT (ms)	225.73 ± 36.37	197.46 ± 29.55	165.45 ± 25.93	272.35 ***

*** *p* < 0.001.

**Table 4:** Mean nGRF and temporal parameters for CMJs. JH = Jump height; BW = Body weight.

Parameter	Mean (±SD)
FzTO (BW)	0.78 ± 0.45
FzLan (BW)	1.77 ± 0.54
tFzLan (BW/s)	57.85 ± 14.77
JH (cm)	33.65 ± 5.92

## Data Availability

The data presented in this study are available on request from the corresponding author. The data are not publicly available due to commercial privacy policy.
